# Associations between sleep and the gut microbiome in adults with colorectal cancer and their caregivers

**DOI:** 10.1038/s41598-025-18402-2

**Published:** 2025-09-22

**Authors:** Jennifer J. Barb, Lena J. Lee, Ayaan Ahmed, Elisa H. Son, Shubhi Nanda, Li Yang, Yuguang Ban, Amanda Ting, Thomas C. Tsai, Youngmee Kim

**Affiliations:** 1 Translational Biobehavioral and Health Promotion Branch, Clinical Center, National Institutes of Health, Bethesda, MD USA; 2 University of Miami, Coral Gables, FL USA

**Keywords:** Sleep disturbance, Gut microbiome, Colorectal neoplasms, Caregivers, Patient-caregiver dyad, Outcomes research, Cancer genomics

## Abstract

Adults with cancer and their caregivers have elevated levels of sleep disturbances. In this cross-sectional exploratory study, we examined associations between subjective sleep indices and gut microbiome features among patients with colorectal cancer (CRC) and their sleep-partner caregivers. Forty participants (20 patient-caregiver dyads) completed sleep diaries, stool sampling, and dietary intake questionnaires individually. Patients and caregivers had comparable demographics, dietary and sleep indices. However, patients had significantly different beta diversity (*p* = .005) and alpha diversity (Inverse Simpson: *p* = .029) as well as 7 more and 6 less differentially abundant taxa compared with their caregivers. Furthermore, only patients categorized with high sleep efficiency (SE) (≥ 85%) also had higher gut microbial diversity (Inv Simpson: *p* = .019 and Shannon Index: *p* = .035). Additionally, some oral and gut microbes were differentially abundant in patients between patients with high or low SE scores. Overall, this study highlights a link between sleep health and gut microbiome characteristics in patients with CRC. Further research is warranted to replicate the current findings with a larger sample, which will allow for the identification of pathways linking sleep to gut microbiome and the development of proper psychobehavioral sleep interventions for adults with cancer and their caregivers to ultimately improve their overall well-being.

**Supplementary Information:**

The online version contains supplementary material available at 10.1038/s41598-025-18402-2.

## Introduction

Patients with cancer experience a myriad of symptoms, including sleep disturbance^1^. Sleep disturbance includes difficulty falling asleep, difficulty staying asleep, and/or sleeping for a nonoptimal duration, resulting in poor sleep efficiency (SE)^2^. Sleep disturbance is highly prevalent across all cancer sites/types and the cancer trajectory (33–40%, as opposed to 15–20% in the general population)^1,3,4^. Sleep disturbance in patients with cancer has been viewed as a treatment-related symptom along with other cytokine-induced sickness behaviors, including fatigue, depression, and pain^5^.

In addition, sleep disturbance is highly common in family caregivers of cancer patients^6^: 36–95% of caregivers either self-reported or displayed objective assessed sleep disturbance, and 4 in 10 caregivers reported at least one sleep-related problem^7,8^. A systematic review concluded that over 72% of caregivers of patients with advanced cancer reported moderate to severe levels of sleep disturbance and an average sleep duration of 4.5 h, which was about a 44% reduction in total sleep time compared with the recommended 8 h^9^. The rate and severity of sleep disturbance in caregivers of patients with cancer are also higher than those in caregivers of patients with other diseases^10–18^ and in demographically similar healthy adults^12^.

Sleep and cancer are shared (dyadic) experiences between adult patients with cancer and their sleep-partner caregiver. Sleep patterns are likely to influence one another. Indeed, a study supported such an assumption illustrating significant correlations between patients with cancer and their sleep-partner caregivers, concurrently (Kendall’s tau-c = 0.301, *p* = 0.02) on indicators of poor sleep quality, operationalized as scores greater than 5 on the Pittsburgh Sleep Quality Index (PSQI) and short sleep duration^19^. Such correlation was also observed longitudinally: patient’s sleep quality at 2 months was positively associated with caregiver’s sleep quality at 4 months (*r* = 0.68, *p* = 0.01), and patient’s longer sleep latency at 2 months was associated with caregiver’s longer sleep latency at 4 months (*r* = 0.28,* p* = 0.05)^19^. Another study assessing 280 dyads with various cancers and symptoms showed that the caregiver burden is influenced by the symptoms of the patients with cancer and that the interdependences of the associations exist at the start of cancer treatment for the patients^20^. These findings suggest that sleep disturbances in adults with cancer and their family caregivers are interrelated, indicating a reciprocal influence on each other’s sleep patterns over time^19,20^.

In recent years, there has been a large focus on clinical research on the associations of sleep disturbance with the gut microbiome^21^. The gut microbiome has been shown to impact overall health by influencing interactions with the immune system, digestion, gastrointestinal health, and mental well-being through metabolites produced by gut bacteria^22^. Disruptions in the gut microbial ecosystem, such as imbalances in the composition of gut bacteria or alterations in microbial diversity, have been associated with far-reaching consequences for ill health, including numerous morbid conditions such as cancer^23^. Growing evidence suggests that the gut microbiota may mediate the relationship between sleep dysfunction or disrupted circadian rhythm and health outcomes^24,25^. However, the respective association of sleep disturbance with the gut microbiome has been limited to individuals with severe levels of sleep disturbance^24,25^.

Sleep disturbance in patients with cancer may also be associated with markers of disrupted gut microbial health or dysbiosis. A study of patients with rectal cancer found an association between sleep disturbance and lower Shannon diversity score (gut microbiome alpha diversity measure)^26^. Another study of patients with breast cancer found positive associations between sleep quality and functional taxa abundances; patients with higher sleep quality had lower relative abundance of *Firmicutes* and higher relative abundance of *Bacteroidetes* at the phylum level, and higher relative abundance and lower relative abundance of *Acidaminococcus* of several genera^27^. Sleep quality and quantity have also been associated with the gut microbiome^28–30^. For example, sleep duration, SOL and sleep efficiency variability have been associated with certain gut genera (e.g. *Faecalibacterium*, *Bacteroides)*, as well as subjective sleep quality related to beta diversity^28–30^. To date, only a few studies with patients with cancer and their caregivers have reported sleep characteristics associated with gut microbiota markers and this work builds upon preliminary results presented at the SLEEP 2024 conference in Houston, Texas^31^. This cross-sectional, exploratory research is aimed to compare sleep indices and gut microbiome features between patients diagnosed with colorectal cancer (CRC) and their sleep-partner caregivers.

## Methods

### Sample participants and procedure

This study employed an observational, cross-sectional design, which included a subset of the data drawn from a longitudinal study focusing on examining the effects of stress regulation on health outcomes in patients with CRC and their spousal caregivers. Written informed consent was obtained from all participants before data collection. All procedures were performed in accordance with the Institutional Review Board of the University of Miami and the research protocol complied with all relevant human research guidelines. All experimental protocols were approved by the University of Miami Institutional Review Board: 20,160,736. This study is comprised of 20 patient-caregiver dyads (N = 40). Dyads were recruited from oncology clinics in Miami, Florida, and data were collected between January and August of 2020. Eligibility criteria for patients were 18 years or older, newly diagnosed with stage I to IV colon or rectal cancer within the past 10 months at the time of enrollment, and who had a partner who shared daily activities, including sleep. Eligibility criteria for caregivers were 18 years or older and a sleep-partner of the patient. Both patients and caregivers were able to read and speak English or Spanish at least at the 5th grade level. Exclusion criteria for both patients and caregivers were (a) active yet untreated psychosis, dementia, substance dependence, phobia pertaining to medical treatment procedure, and suicidal ideation in the past year; (b) active yet untreated sleep apnea, narcolepsy, and restless leg syndrome (sleep apnea: 3 on the Berlin Questionnaire without the use of positive airway pressure machines during sleep; narcolepsy: >  = 14 on the Ullanlinna Narcolepsy Scale^32,33^, (c) inability to see or hear; (d) poor cognitive functioning status (< = 24 on the Mini Mental State Examination^34^; or (e) patients who are under end-of-life care or endorsed poor physical functioning status (< = 50 on the Karnofsky Performance Status and >  = 3 on the Eastern Cooperative Oncology Group Performance Status;^35,36^. Cancer treatment status was unknown for 1 patient; only 3 patients were in active cancer treatment during the time of the study. Patients were diagnosed approximately 7 months (N = 7), 1.5 years (N = 11), and 2.5 years (N = 2) prior to participating in this study. Educational status was grouped into low (high school diploma/GED or less) or high (technical/vocational school, some college, associate degree or higher).

Participants filled out a one-time questionnaire about their sociodemographic characteristics and completed daily sleep diaries for 14 consecutive days. Participants provided two stool samples approximately 10 days apart. Additionally, participants completed a web-based dietary assessment on the day of stool sample collection. Written informed consent was obtained from all participants before data collection. All procedures were performed in accordance with the Institutional Review Board of the University of Miami.

### Sleep indices

Sleep diaries were completed by participants in the morning upon waking using the Consensus Sleep Diary^37^. Sleep duration, defined as the total number of hours spent sleeping, was quantified by the duration between “[after getting into bed] What time did you try to go to sleep?” and “What time was your final awakening?”. Sleep onset latency (SOL) is defined as minutes between intending to sleep and sleep onset. Waking after sleep onset (WASO), defined as minutes awake between sleep onset and final awakening, was quantified by the total number of minutes reported for the item, “If [woke up] more than once, in total, how long were these awakenings?”. Total time in bed is described as the total time spent in bed with the intention of sleeping (e.g., “Time of final awakening—time of initial sleep attempt”). Sleep efficiency (SE) was calculated by [total time spent asleep (Sleep Duration: SD)—time between trying to and actually falling asleep (Sleep Onset Latency: SOL)—time awake after sleep onset and before final awakening (Wake After Sleep Onset)] divided by total time in bed with the intention of sleeping. In other words, SE is the percentage of time spent asleep while in the bed intending to sleep. Sleep efficiency was evaluated in this study using the continuous value of percent of efficiency score and also as a dichotomized variable using previously established cutoffs for dichotomization as follows: ‘low (SE < 85%)’ or ‘high (SE: ≥ 85%)’^38^.

### Dietary intake indices

Participants completed a web-based version of the 2018 version of the Automated Self-Administered 24-h Dietary Assessment Tool (ASA24) as their dietary assessment. To support data accuracy and comprehension across varying literacy and education levels, study personnel were available to assist all participants during completion of the ASA24, either in person or by phone. This assistance was particularly important for ensuring data collection quality among participants. Total macronutrients, including carbohydrates, protein, fat, and the percent of energy from carbohydrates, protein, and fat, as well as total energy (kcal), alcohol intake, and daily fiber (g) were calculated per individual. Diet quality was assessed using the Healthy Eating Index-2020 (HEI-2020)^39^. Average dietary intake indices were calculated from the daily totals across days for each participant.

### Stool sample collection

Stool samples were collected from participants using provided kits that included two Fecotainer® kits, dry-ice packs, gloves, and a cooling bag. Participants were instructed to collect the stool samples in the morning (first bowel movement of the day) and to store the sample either in their home refrigerator (at 4C) or in the insulated bag with dry ice. To minimize in-person contact during the COVID-19 pandemic, all materials (e.g., collection kits, gloves, labeled containers) were delivered and returned via courier or drop-off at designated collection points. Study staff adhered to institutional biosafety protocols and COVID-19 safety guidelines during all stages of handling and processing, including the use of personal protective equipment (PPE) and surface decontamination procedures. This flexibility was provided to accommodate participant comfort and household logistics, particularly in the context of the COVID-19 pandemic. Stool depositions were collected in a Fecotainer (Fisherbrand Commode Specimen Collection System, Fisher Scientific, Inc.), which was kept in an insulated cooling bag in below 40° F (4 °C) until shipped to the study lab. All stool samples were aliquoted in 5 ml centrifuge tubes with caps to be stored in − 80 °C freezer until sequencing.

### Gut microbiome sample preparation and sequencing

Shotgun metagenomics sequencing was performed at the University of Minnesota Genomics Center (https://genomics.umn.edu/). DNA quantification was performed using a fluorimetric PicoGreen assay and purity was assessed through Nanodrop. For a sample to pass quality control methods (QC), it had to be greater than 0.2 ng/ul. Quality controlled samples were submitted to TruSeq NexteraXT DNA library preparation steps. Illumina sequencing libraries using Illumina’s NexteraXT DNA Sample Preparation Kit (Cat. # FC-131-1096) were generated from genomic DNA samples. In summary, 1 ng of gDNA is simultaneously fragmented and tagged with a unique adapter sequence. This “tagmentation” step is mediated by a transposase. The tagmented DNA is simultaneously indexed and amplified for 12 PCR cycles. Final library size distribution was validated using capillary electrophoresis and quantified using fluorimetry (PicoGreen). Pooled libraries were denatured and diluted to the appropriate clustering concentration. Sequencing libraries were loaded onto a NovaSeq paired-end flow cell, and on-instrument clustering was performed. Sequencing was then initiated using Illumina’s 2-color sequencing-by-synthesis (SBS) chemistry. Following completion of Read 1, two separate index reads (8 or 10 base pairs each) were performed to capture sample barcodes. The clustered library fragments were then re-synthesized in the reverse direction to generate the template for Read 2. Base call (.bcl) files for each cycle of sequencing were generated by Illumina Real Time Analysis (RTA) software. The base call files and run folders were streamed to servers maintained at the Minnesota Supercomputing Institute. Primary analysis and de-multiplexing were performed using Illumina’s bcl2fastq v2.20, a software for converting sequencing data into BCL or FASTQ files. The final result of the bcl2fastq workflow was de-multiplexed FASTQ files that were released to client accounts for subsequent analysis by the mapping software and aligner of their own choosing.

### Gut microbiome bioinformatic methods

#### FASTQ file processing and taxonomic classification

Shotgun sequence reads were trimmed and filtered, and host DNA contaminated reads were removed using KneadData workflow (https://huttenhower.sph.harvard.edu/kneaddata/). Metagenomic profiling of microbial abundance and taxonomy assignment was performed with MetaPhlAn (ver.2)^40^, which uses clade-specific marker genes from 17,000 reference genomes. Species level taxa were filtered to remove low abundance and prevalence taxa. Species level counts had to have at least a count of 10 or greater in at least 60% of the averaged samples to be included for statistical analysis comparisons. This filter removes low abundance and low prevalence taxa. Functional composition and abundance were determined using HUMAnN2^41^ with the complete UniRef gene family^42^, KEGG functional modules and pathways ^43–45^, and MetaCyc metabolic pathway databases^46^. Gene abundance was calculated in read per kilobase (RPK), and pathway abundance was obtained after computing the sum over abundances of pathway-associated genes. Alpha diversity was calculated, and the following 3 metrics were reported: Chao1, Shannon, Inverse Simpson indices. Multi-dimensional scaling analysis plots were used for beta diversity assessment using Bray–Curtis distance, and the associations between patients and caregivers were analyzed based on permutational analysis of variance (PERMANOVA) test using methods provided by the phyloseq^47^ and vegan^48^ Bioconductor packages. Functional composition data was filtered requiring that at least 40% of the averaged samples have an abundance of 100 or more. The centered log ratio (CLR) with a parameterization transformation to account for zeros was computed on the taxa counts (https://github.com/thomazbastiaanssen/deleuze). Differential abundance testing using Wilcoxon Signed Rank testing procedures of the paired data was performed on centered-log-ratio-transformed abundance values. Gut microbial alpha diversity indices and taxa abundance data from each participant’s replicate stool sample were averaged, and the averages were used as descriptive data and for statistical testing.

### Statistical analysis

IBM SPSS version 29.02.2 and JMP version 16 Statistical Computing Software (SAS Headquarters, Cary, NC) were utilized for visualization and statistical testing procedures. Descriptive statistics (mean and standard deviation for continuous data, frequencies and percentages for categorical data) were computed for all study variables for patients and caregivers. Differences in demographics, dietary and sleep indices between patients and caregivers were tested using paired non-parametric testing procedures (Wilcoxon signed rank test for continuous data and McNemar test for nominal data). Alpha diversity features including Chao1, Shannon, and Inverse Simpson indices were compared between patients and caregivers using paired non-parametric testing procedures. To assess potential associations between dietary intake and gut microbial diversity, we conducted within-group exploratory analyses using Spearman’s rank correlation coefficients. Correlations were calculated separately for patients with colorectal cancer and their sleep-partner caregivers to examine relationships between dietary quality measures (HEI-2020) and total dietary fiber intake (g/day) and alpha diversity metrics, including the Shannon diversity index, Chao1, and Inverse Simpson index. Spearman correlation was chosen due to its nonparametric robustness and suitability for small sample sizes and non-normally distributed data.

Bray Curtis dissimilarity, assessing gut microbial composition differences, was assessed between patients and caregivers using PERMANOVA. Differential gut taxa abundances between patients and caregivers were investigated using a Wilcoxon signed rank testing procedure with the Benjamini–Hochberg multiple comparisons correction significance level set at > 20% false discovery rate (FDR)^49^. Log fold change (LFC) refers to differential fold-change abundances between patients and caregivers, which were calculated using the central log ratio (CLR) transformed data. Spearman correlation tests were performed to examine the associations of sleep indices with gut microbiome features, including alpha diversity measures and CLR transformed taxa abundances within patients and caregivers separately. Mann–Whitney U test was used to test the differences of gut microbial features between dichotomized sleep efficiency groups (*low* vs. *high*) within patients and caregivers separately. In addition, only for patients, a Mann–Whitney U test was used to examine whether active cancer treatment status (active vs inactive cancer treatment) was associated with gut microbial diversity measures. Statistical significance was determined by a 2-tailed *p* value < 0.05 and for gut microbial features, the adjusted significance level was set at 20% FDR.

## Results

### Sample characteristics

Sociodemographic and clinical characteristics of this study sample (N = 40; 20 dyads) are shown in Table 1. No significant differences between patients and caregivers in the demographics and BMI were observed. Participants were primarily middle-aged, Hispanic, employed, overweight, and had some college education. Patients were 60% female, had an average age of 54.63 ± 9.22, and an average body mass index (BMI) of 27.30 ± 5.47. Furthermore, 55% (N = 11) of patients were employed and 85% (N = 17) had a high level of education. Distribution of cancer stage diagnosis among patients was as follows: 45% (N = 9) with stage I and II and 55% (N = 11) with stage III and IV CRC. Three patients were actively undergoing chemotherapy at the time of the study collection. Caregivers were 60% male with an average age of 56.34 ± 11.19, and an average BMI of 29.80 ± 5.31. Additionally, 70% (N = 14) of the caregivers were employed with, and 90% (N = 18) had a high level of education. Thirty-three participants (N = 16 patients; N = 17 caregivers) completed dietary recalls. Descriptive averages of diet quality and dietary intake indices for patients and caregivers are shown in Supplemental Table 1 and no significant differences were observed between patient and caregiver among any of the dietary intake indices assessed.

**Table 1:** Sociodemographic and clinical characteristics of sample.

	All N = 40	Patients N = 20	Caregivers N = 20	Test statistic	*P* value^a^
Mean (SD) and range or N (%)					
Age (years)	54.89 (9.76), 34.31–77.13	54.63 (9.22), 38.81–74.50	55.15 (10.51), 34.31–77.13	S = 28.0	.312
BMI (kg/m^2^)	28.55 (5.53), 18–43	27.30 (5.55), 18–40	29.80 (5.38), 22–43	S = − 37.5	.165
N (%)					*P* value^b^
Gender (female)	20 (50.0%)	12 (60.0%)	8 (40.0%)	*Χ*^*2*^ = *0 .80*	.371
Race/ethnicity					
Non-hispanic black	4 (10.0%)	2 (10.0%)	2 (10.0%)	*Χ*^*2*^ = *1.31*	.933
Non-hispanic white	11 (27.5%)	5 (25.0%)	6 (30.0%)		
Hispanic	25 (62.5%)	13 (65.0%)	12 (60.0%)		
Employment status^c^					
Employed	25 (62.5%)	11 (55.0%)	14 (70.0%)	*Χ*^*2*^ = *1.28*	.256
Unemployed	15 (37.5%)	9 (45.0%)	6 (30.0%)		
Education level^d^					
Low	5 (12.5%)	3 (15.0%)	2 (10.0%)	*Χ*^*2*^ = *0.33*	.564
High	35 (87.5%)	17 (85.0%)	18 (90.0%)		
On cancer treatment	–	3 (15%)	–	–	–
Cancer stage					
Non-advanced (I-II)		9 (45.0%)		–	–
Advanced (III-IV)		11 (55.0%)		–	–

^a^Wilcoxon Signed Rank Test; ^b^Chi-Square Test. ^c^Employment Status: Employed = full-time or part-time employed, or self-employed; Unemployed =  = not employed. ^d^Education: low =  = high school diploma/GED or less; high = technical/vocational school, some college, associate degree or higher. Abbreviations: BMI = Body Mass Index; SD = Standard Deviation.

### Comparisons of sleep indices between patients and caregivers

As shown in Table 2, patients had comparable levels of sleep indices with their caregivers. On average, participants spent about 9 h in bed and were asleep for 8.5 of those hours. Sleep duration was slightly outside of the optimal range of 7–8 h of sleep, with most patients (85%) and caregivers (90%) falling outside of this. In addition, for other sleep indices, participants had normative levels of sleep onset latency (SOL), wake after sleep onset (WASO), and sleep efficiency (SE). Similarly, only a few participants showed suboptimal levels of sleep patterns. Specifically, 2 patients had suboptimal SOL (> 20 min), and 2 participants (1 patient and 1 caregiver) had suboptimal WASO (> 20 min). Twenty % of patients (N = 4) and 25% of caregivers (N = 5) had suboptimal (or low) SE (< 85%).

**Table 2:** Sleep indices across the whole sample and between patients and caregivers.

Sleep indices	All (N = 40)	Patients (N = 20)	Caregivers (N = 20)	Test statistic	*P* value^a^
	Mean (SD)				
Time in Bed (h)	9.23 (1.47)	9.35 (1.51)	9.12 (1.46)	S = 13.0	.641
Sleep Duration (h)	8.51 (1.37)	8.17 (1.20)	8.85 (1.42)	S = − 44.0	.105
Sleep Onset Latency (m)	5.13 (6.94)	5.73 (8.72)	4.54 (4.71)	S = 4.0	.890
Wake after Sleep Onset (m)	3.19 (6.91)	3.31 (6.49)	3.08 (7.47)	S = 16.0	.574
Sleep Efficiency (SE)	.888 (.01)	.872 (.12)	.904 (.07)	S = − 34.5	.205
High or Low SE	N (%)				*P* value^b^
Low or < 85%	9 (20%)	4 (20%)	5 (25%)	Χ^2^ = .126	.723
High or ≥ 85%	31 (80%)	16 (80%)	15 (75%)		

^a^Wilcoxon Signed Rank Test. ^b^Chi-Square Test; Abbreviations: h = hours, m = minutes.

### Investigating gut microbiome features of patients with colorectal cancer and their caregivers

A total of 80 fecal samples were submitted for gut microbiome shotgun metagenomics sequencing. The average mapped reads across all samples were 33,292,434 ± 5,535,927. Overall, the gut microbiome over this cohort was represented with 14 phyla, 211 genera, and 613 species. Low abundance and low prevalence filtering removed 474 species level taxa, which allowed for 139 taxa to be investigated for further analysis.

**Fig. 1:**
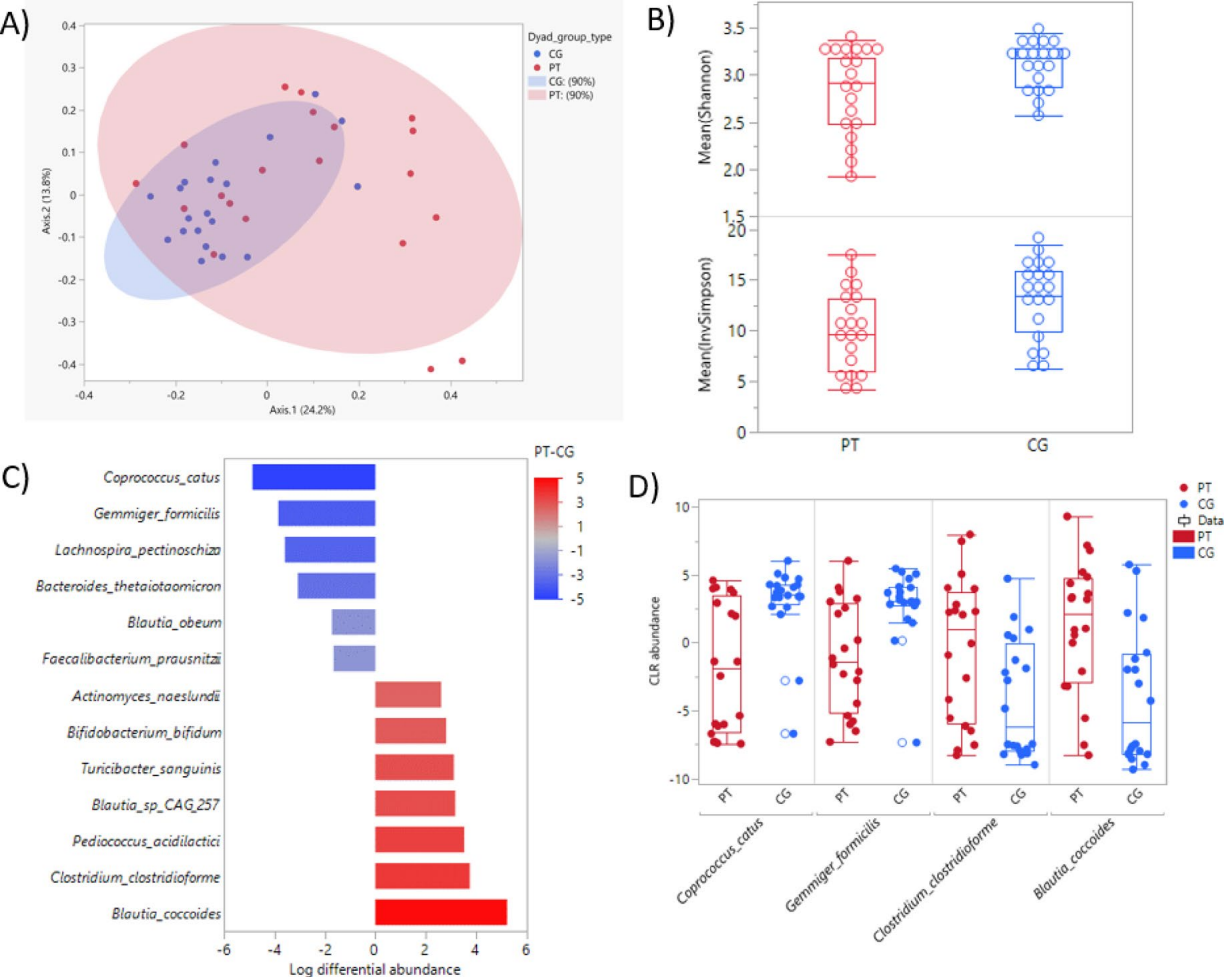
Gut microbiome features of caregivers and patients. (**A**) Principal Coordinate Analysis based on Bray–Curtis dissimilarity of gut compositional differences (R-squared: 0.070, *p* = .005). (**B**) Box-and-whisker plot of alpha diversity measured by Shannon Diversity Index (*p* = .058) and Inverse Simpson Diversity Index (*p* = .030) between patients and caregivers. (**C**) Differentially abundant taxa between patients and caregivers by log differential abundance (x-axis) (*p* < .05 and 20% FDR). Heat map gradient indicates log fold change difference. (**D**) Box plot of the two most abundant species and the two least abundant species in patients. Red: patients (PT); Blue: caregivers (CG).

A significant difference between patients and caregivers of the gut microbial compositional structure (assessed by Bray–Curtis Dissimilarity PERMANOVA) was observed (R-squared: 0.070, *p* = 0.005) (Fig. 1A). Furthermore, patients had lower gut microbial alpha diversity assessed by the Inverse Simpson Index than caregivers (*p* = 0.030) (Fig. 1B). However, other alpha diversity measures assessed showed no significant differences between patients and caregivers (Shannon Diversity: *p* = 0.058 and Chao1: *p* = 0.227) (Table 3). Among patients, chemotherapy treatment status (Supplemental Table 2) and cancer stage (Supplemental Table 3) showed no significant differences of alpha diversity indices. As an exploratory analysis, we examined correlations between alpha diversity, diet quality and fiber intake (HEI-2020 scores and fiber intake) within each group. Among patients with CRC, higher HEI-2020 scores were significantly associated with greater microbial diversity, as measured by the Shannon index (Spearman ρ = 0.464, *p* = 0.006) and the Inverse Simpson index (Spearman ρ = 0.405, *p* = 0.018). No significant correlations were observed between HEI-2020 and diversity indices among caregivers, nor were fiber intake measures significantly associated with any alpha diversity metric in either group (Supplemental Table 4).

**Table 3:** Alpha diversity between patients and caregivers.

Alpha diversity indices					
Alpha diversity measures	All N = 40	Patients N = 20	Caregivers N = 20	Statistic	*P* value^a^
Mean (SD), Range					
Shannon	2.95 (0.38) 1.92–3.44	2.80 (0.44), 1.92–3.38	3.10 (0.25), 2.57–3.44	S = 51.00	.058
Inverse Simpson	11.38 (4.00) 4.15–18.44	9.88 (3.85), 4.15–17.48	12.89 (3.65), 6.18–18.44	S = − 58.00	**.030**
Chao1	159.18 (19.29) 119.50–210	155.25 (19.30), 119.50–187	163.1 (18.93), 126.50–210	S = − 33.00	.227

^a^Wilcoxon Signed Rank Test; Observed features are reported at the species level. Significant values are in bold.

**Table 4:** Associations between alpha diversity with sleep measures within patients and caregivers.

Alpha diversity measures	Patients		Caregivers	
	Spearman’s ρ	*P* value^a^	Spearman’s ρ	*P* value^a^
Sleep Efficiency				
Inverse Simpson	.443	**.051**	.010	.967
Chao 1	− .108	.652	.149	.531
Shannon	.413	.071	.045	.852
Sleep Duration				
Inverse Simpson	.050	.835	.139	.558
Chao 1	.058	.807	.317	.174
Shannon	.072	.762	.108	.649
Time in Bed				
Inverse Simpson	− .378	.101	.117	.624
Chao 1	.059	.806	.261	.266
Shannon	− .304	.193	.087	.715
Sleep Onset Latency				
Inverse Simpson	− .093	.696	− .154	.516
Chao 1	.032	.893	− .165	.486
Shannon	− .090	.705	− .284	.225
Wake after Sleep Onset				
Inverse Simpson	− .119	.616	.027	.910
Chao 1	.209	.377	− .029	.903
Shannon	− .066	.783	− .069	.774

^a^Spearman Correlation Test. Significant values are in bold.

Of the 139 species level taxa included for analysis, the most abundant phyla were *Firmicutes* (61.94% in patients, 61.69% in caregivers) and *Actinobacteria* (25.14% in patients, 28.79% in caregivers) (Supplemental Fig. 1A). Individual relative abundances of the filtered species level taxa across all participants are shown in a stacked bar plot in Supplemental Fig. 1B. The most abundant species of the entire cohort were *Faecalibacterium prausnitzii* (7.10% ± 6.88), *Collinsella aerofaciens* (6.69% ± 7.99), *Eubacterium rectale* (5.57% ± 7.32), *Fusicatenibacter saccharivorans* (4.79% ± 5.50), *Bifidobacterium longum* (4.75% ± 5.50), and *Bifidobacterium adolescentis* (3.94% ± 7.49).

Patients and caregivers showed 13 taxa to be differentially abundant (*p* < 0.05 and 20% FDR), with seven of those less abundant in patients and six more abundant in patients (Fig. 1C) (Supplemental Table 5). Among these taxa that showed differential abundance, *Coprococcus catus* (*p* < 0.001 and 6% FDR, LFC:4.87) and *Gemmiger formicilis* (*p* < 0.001 and 4% FDR, LFC: − 3.85) had the highest significant abundance in caregivers (lowest in patients), whereas *Clostridium clostridioforme* (*p* < 0.001 and 9% FDR, LFC: 3.74) and *Blautia coccoides* (*p* < 0.001 and 13% FDR, LFC: 5.22) had the highest significant abundance in patients (lowest in caregivers) (Fig. 1D). The HUMAnN2 workflow, for which bacterial genomic functional potential was measured using the Kyoto Encyclopedia of Genes and Genomes (KEGG) pathway identifiers (see Methods), revealed 468 KEGG pathways. After filtering to remove low abundance and low prevalence abundance pathways, 267 KEGG identifiers were retained for further analysis. Testing differential abundance of KEGG pathways between patients and caregivers revealed 13 KEGG pathways that were differentially abundant at an unadjusted *p* < 0.05 but none were significant at 20% FDR. (Supplemental Fig. 2A and B, Supplemental Table 6).

**Table 5:** Associations of sleep efficiency (continuous or categorical) with taxa among patients.

Species	Spearman correlation with SE			High/Low SE		
	ρ	*P* Value^a^	FDR	Z-Statistic	*P* Value^b^	FDR
*Actinomyces oris*	− 0.805	< .001*	**0%**	2.787	.005*	**18%**
*Streptococcus mitis*	− 0.735	< .001*	**2%**	2.787	.005*	**18%**
*Atopobium rimae*	− 0.407	.075	39%	2.976	.003*	**18%**
*Atopobium parvulum*	− 0.640	.002*	**7%**	2.976	.003*	**18%**
*Actinomyces sp. oral taxon 181*	− 0.636	.003*	**7%**	2.693	.007*	**20%**
*Dorea formicigenerans*	0.612	.004*	**8%**	− 2.315	.021	24%
*Lactobacillus rogosae*	0.601	.005*	**9%**	− 2.315	.021	24%
*Streptococcus parasanguinis*	− 0.702	< .001*	**3%**	2.504	.012	24%
*Actinomyces graevenitzii*	− 0.589	.006*	**9%**	2.409	.016	24%
*Streptococcus vestibularis*	− 0.588	.006*	**9%**	1.464	.143	52%
*Streptococcus salivarius*	− 0.615	.004*	**8%**	1.275	.202	60%
*Lactococcus lactis*	− 0.532	.016*	**19%**	1.275	.202	60%
*Paludisphaera borealis*	0.529	.016*	**19%**	− .1417	.887	94%

* indicates FDR < 20%; Abbreviations: FDR = False Discovery Rate. ^a^Spearman correlation test. ^b^Mann Whitney U test. Significant values at ≤ 20% FDR are in bold.

### Investigating the associations of sleep indices with gut microbial features

**Fig. 2:**
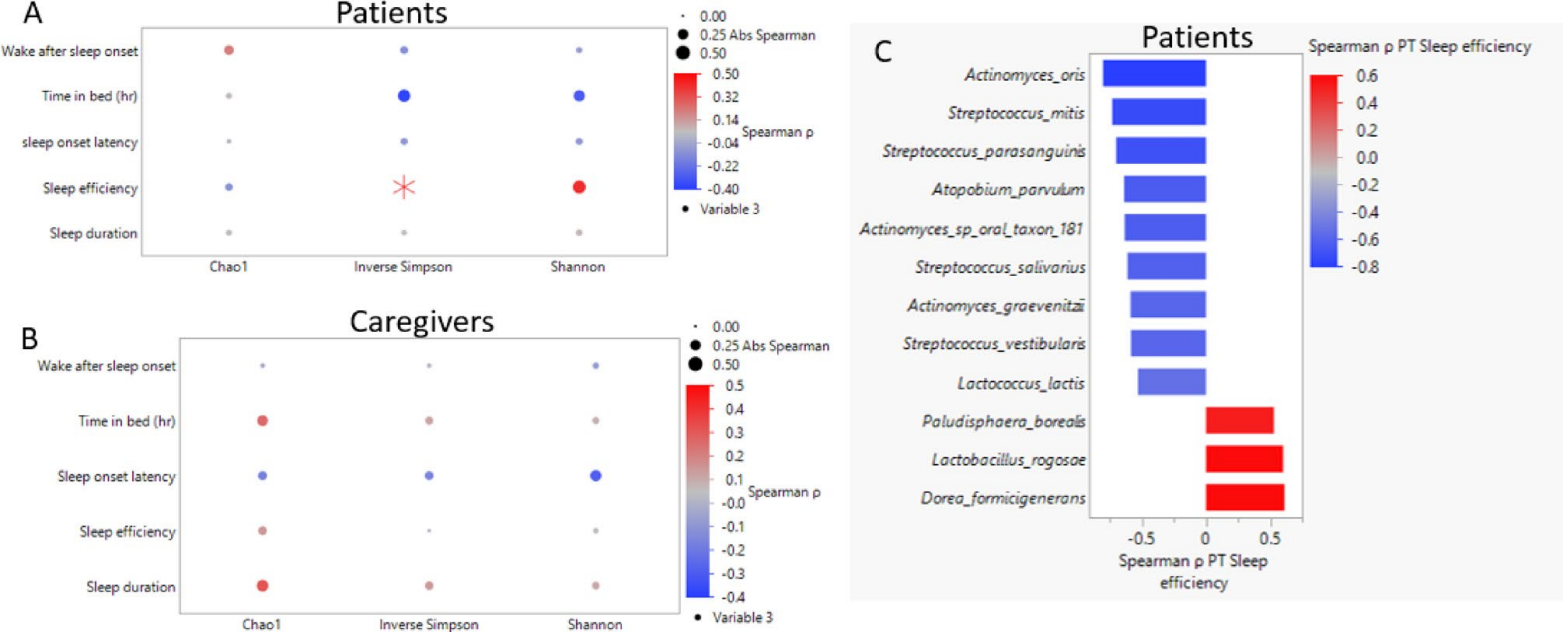
Associations of sleep indices with gut microbial features among patients and caregivers. Spearman correlation dot plot with sleep indices (y-axis) and alpha diversity indices (x-axis: Chao1, InverseSimpson, Shannon) of the gut microbiome for (**A**) Patients and (**B**) Caregivers. Dot color indicates Spearman Correlation coefficient and size indicates absolute value of the Spearman ρ. (*p < 0 .05). (**C**) Twelve taxa significantly correlated with SE scores within patients (*p* < .05; 20% FDR). Spearman correlation coefficient (x-axis). Heat map gradient indicates Spearman correlation coefficient.

The relationship between sleep indices and gut microbial alpha diversity was explored separately for patients (Fig. 2A) and caregivers (Fig. 2B) (Table 4). Among patients, continuous SE scores showed a trend toward a positive association with alpha diversity as measured by Inverse Simpson index (*p* = 0.051). No other significant associations were observed among patients or caregivers across any alpha diversity metrics (p > 0.05). Additionally, among caregivers, sleep indices were not correlated with gut microbial diversity nor were any trends observed. When correlations were assessed at the taxa level, 12 taxa were found to be significantly correlated with the continuous SE scores within patients only (*p* < 0.05 and > 20% FDR) (Fig. 2C, Table 5, Supplemental Fig. 3). Of the 12 taxa, SE was most negatively correlated with three taxa: *Actinomyces oris* (Spearman ρ = − 0.80, *p* < 0.001, FDR 0%), *Streptococcus mitis* (Spearman ρ = -0.73, *p* < 0.001, FDR 2%), and *Streptococcus parasanguinis* (Spearman ρ = − 0.70, *p* < 0.001, FDR 3%), whereas SE was most positively correlated at a 10% FDR or less with two taxa: *Dorea formicigenerans* (Spearman ρ = 0.61, *p* = 0.004, FDR 8%) and *Lactobacillus rogosae* (Spearman = 0.60, *p* = 0.005, FDR 9%). Among caregivers, no taxa were found to be significantly correlated with continuous sleep indices.

**Fig. 3:**
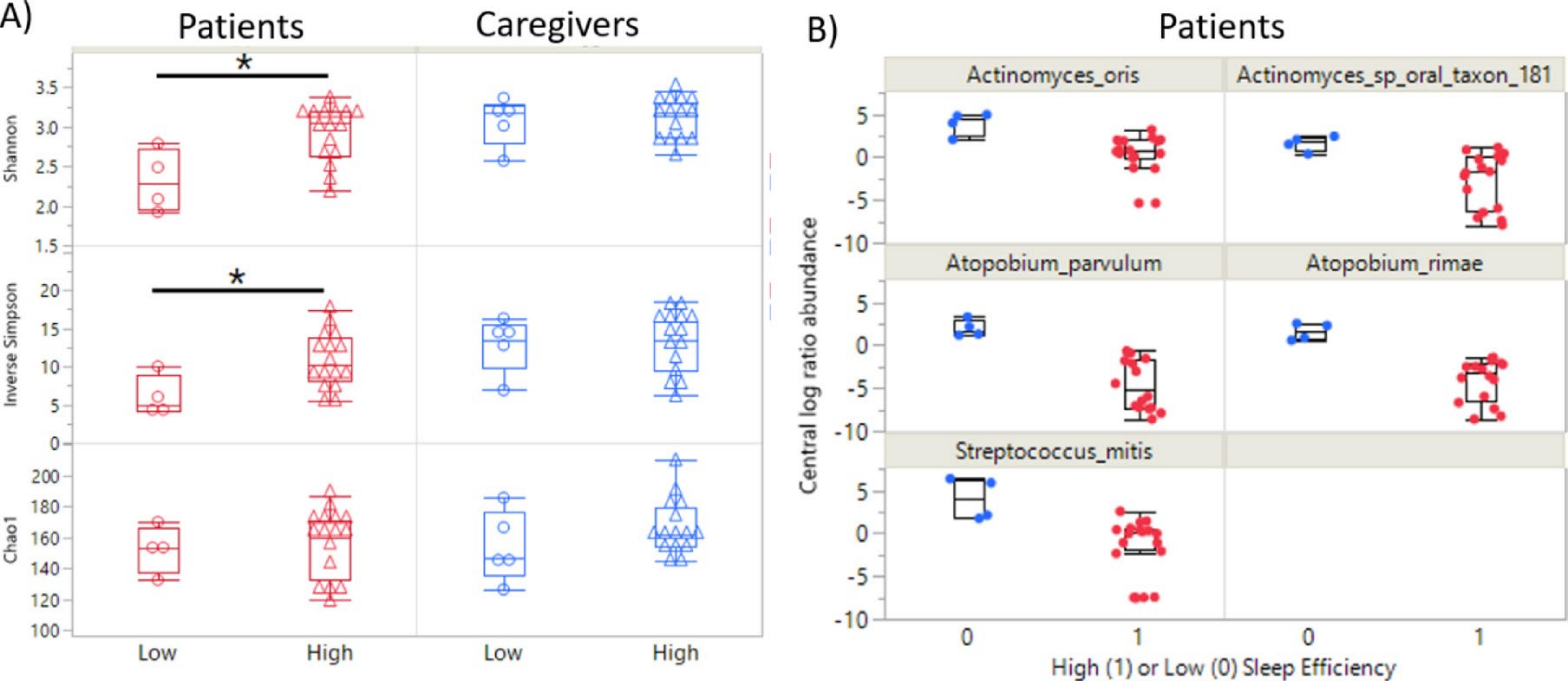
Alpha diversity and differential abundance between individuals grouped as high or low sleep efficiency. (**A**) Box plot alpha diversity indices between high and low SE groups within patients and caregivers. * indicates *p* < 0.05. (**B**) Patient differential CLR abundances (y-axis) of five taxa significantly different between high (1) and low (0) SE.

Gut microbial genomic potential (indicated by KEGG pathways) showed that five pathways were associated with sleep indices for both patients and caregivers at *p *< 0.05 and 20% FDR (Supplemental Table 7). Specifically, among patients, SOL was negatively correlated with super pathway of menaquinol-9 biosynthesis (Spearman ρ: − 0.807, *p* < 0.001, FDR: 4%) and super pathway of menaquinol-10 biosynthesis (Spearman ρ: − 0.807, *p* < 0.001, FDR: 4%) within caregivers, while patients’ SOL was negatively correlated with pyruvate fermentation to acetone (Spearman ρ: − 0.167, *p* < 0.001, FDR: 5%). In addition, patients’ time in bed was negatively correlated with both pyruvate fermentation to butanoate and super pathway of *Clostridium acetobutylicum* acidogenic fermentation (Spearman ρ: − 0.734, *p* < 0.001, FDR: 5%).

### Investigating the associations of low versus high sleep efficiency with gut microbiota features

The dichotomized sleep efficiency (*low* < 85% versus *high* ≥ 85%) was tested for its associations with gut microbial features. Among patients, low SE was significantly associated with lower alpha diversity, measured by both Shannon (*p* = 0.035) and Inverse Simpson (*p* = 0.019) indices (Fig. 3A, Supplemental Table 5). Among caregivers, the *low* versus *high* SE groups were not significantly related to alpha diversity (*p* > 0.05) (Fig. 3B, Supplemental Table 8). At the taxa level, among patients, 20 taxa were significantly different between the two groups (*p* < 0.05), but only five of those passed 20% FDR (Fig. 3B, Table 5). Those five taxa were found to be significantly depleted in patients with high SE and all were related to the oral microbiome: *Actinomyces oris* (*p* = 0.005 and FDR 18.5%), *Actinomyces sp. oral taxon 181* (*p* = 0.007 and FDR 19.7%), *Atopobium parvulum* (*p* = 0.003 and FDR 18.5%), *Atopobium rimae* (*p* = 0.003 and FDR 18.5%), and *Streptococcus mitis* (*p* = 0.005 and FDR 18.5%). The depletion of the oral bacteria in patients with high sleep efficiency suggests that they may play a role in regulating sleep, or conversely, that sleep efficiency might influence the balance of the microbiome specifically for patients with CRC. Among caregivers, four taxa, primarily part of the gut microbiome within the colon and large intestine, were significantly different between high and low SE (*p* < 0.05), but they did not pass 20% FDR (Supplemental Table 9, Supplemental Fig. 4).

## Discussion

In this study, we compared the sleep and gut microbiome features of adult patients with CRC with those of their sleep-partner caregivers and investigated the associations of sleep indices with gut microbiome features. Patients’ demographics, sleep indices, dietary intake indices, and diet quality were similar to those of their caregivers. Participants also had normative levels of sleep patterns, except for sleep duration. Despite these similarities in patients and caregivers who live together, patients had different gut microbial compositional structure from their caregivers.

Our study showed that patients and caregivers had comparable sleep behaviors, such as sleep onset latency, wake after sleep onset, time in bed, sleep duration, and sleep efficiency. This corroborates previous research indicating that couples tend to have similar sleep behaviors^50^. However, our sample having close to normative levels of sleep patterns is not consistent with existing studies with adult cancer patients^1,3,4,51^. Our sample also showed participants’ dietary patterns were comparable to the Standard American Diet^52^. Similar dietary patterns, which is consistent to existing studies reporting individuals who live together tend to consume similar foods and diets^53–55^. Additionally, exploratory analyses identified significant positive correlations between diet quality and alpha diversity in patients with CRC, suggesting that greater overall diet quality may be associated with increased gut microbial diversity in this population (Supplemental Table 4). These findings align with previous research indicating that dietary patterns influence the gut microbiome and highlight the need for future studies to incorporate dietary quality as a potential modifier or confounder when examining gut microbiome associations^56,57^. Notably, no such associations were observed among caregivers, and fiber intake alone was not significantly correlated with diversity metrics in either group. These results suggest that broader dietary patterns may play a more important role in shaping the microbiome than individual nutrients alone.

Contrary to sleep and dietary patterns, our patients had differentially abundant bacteria from their caregivers: patients had slightly lower gut microbial alpha diversity (measured by the Inverse Simpson index) than their caregivers and had different abundance from caregivers in 13 taxa. The findings are inconsistent with existing studies that showed similarities of gut microbial features among individuals within the same household, especially spousal pairs^58–61^. We can speculate that the differences in gut microbiome features between patients and cohabiting caregivers may be due in part to the impact of colorectal cancer and its treatment on the patients’ gut microbiome^62^. Colorectal cancer evokes significant changes to the gut microbiomes of patients, where past studies have shown significantly lesser gut microbial diversity, compositional structure, and differential bacteria^63^. A recent study by Zhou et al., showed that 20 patients with colorectal cancer reported significantly less alpha diversity in CRC patients compared to the control group and significantly different beta diversity between the groups and that genera such as *Parabacteroides*, *Bacteroides*, and *Dialister* were significantly more abundant in CRC patients in this study^63^. Our patients with colorectal cancer also had lower alpha diversity and differential beta diversity, and 13 differential taxa abundances compared with those of the caregivers. Of the 13 differential taxa, *Coprococcus catus*, *Gemmiger formicilis (G.formicilis), Lachnospira pectinoschiza (L.pectinoschiza)*, *Bacteroides thetaiotaomicron* (*B.thetaiotaomicron*) were found to be significantly depleted (lesser in abundance) in patients. *Coprococcus catus* is a species of anaerobic cocci which produces propionate and butyrate, both of which are beneficial metabolites to human health^64^. *B.thetaiotaomicron* is involved in polysaccharide breakdown. The depletion of these taxa may be implicated in the pathogenesis of cancer^65,66^. Furthermore, the depletion of *G. formicilis* and *L.pectinoschiza* that have protective roles in maintaining gut health may suggest gut microbiome’s alterations in patients with colorectal cancer as^67^. Interestingly, *Faecalibacterium prausnitzii* (*F. prausnitzii)* was significantly depleted in the patients in this study compared to their sleep-partner caregivers, which is consistent with previous research that has reported that *F. prausnitzii* act as an anti-tumorigenic bacterium within the colon to significantly reduce abnormal growths in the lining of the colon in preclinical rat models with colorectal cancer^68^. These results underscore the importance of the gut microbiome composition in colorectal cancer and suggest that further research into the role of beneficial microbes, like *F. prausnitzii,* could provide valuable insights for potential therapeutic strategies at improving patient outcomes*.*

This report is the first to present a comparison of gut microbiome features between patients with CRC and their sleep-partner caregivers while also exploring associations between sleep and the gut microbiota in couples coping with cancer. Among patients only, higher sleep efficiency trended toward a positive association with gut microbial alpha diversity, along with an increased abundance of three taxa, and decreased abundance of nine taxa. These findings are consistent with previous studies demonstrating links between sleep quality and quantity gut microbiome composition^28–30^. For example, previous research has shown associations with certain gut bacteria with sleep duration, SOL and sleep efficiency variability, as well as associations with differential beta diversity and subjective sleep quality^28–30^. In 2019, Smith et al. reported positive correlations between gut microbiome diversity and total sleep time and SE, and they reported negative correlations between gut microbiome diversity and WASO^69^. Wu et al. furthered these results by portraying a causal association between seven sleep-related traits and 68 bacterial taxa^70^. This significant association between the gut microbiome and sleep may be explained by the gut-brain axis, and perturbations in gut microbial homeostasis that can affect overall sleep health^71,72^. In particular, sleep physiology and the gut microbiome are also interconnected through the immune system, as sleep physiology is linked with the cytokines IL-1β and IL-6, and IL-1β and IL-6 production has been shown to be induced by the gut microbiota^69,73^. Our findings help to support this previous work but additional analyses with large sample sizes should be conducted to build upon our knowledge on sleep-gut links in patients with CRC.

This study assessed subjective sleep indices using sleep diaries and we found a trend within patients only where SE scores were slightly associated with alpha diversity which is consistent with an existing population-based study of 720 participants published in 2024^28^. Holzhausen et al., assessed sleep indices using actigraphy methodology where they showed that lower SE scores were also associated with lower microbiome richness and diversity. Our study found 12 taxa to be significantly correlated with patients’ SE and of those, 3 were positively correlated with patients’ higher SE scores. One of those, *Lactobacillus rogosae,* which indicates a healthy gut microbiome, was more abundant in patients with higher SE, also consistent with an existing study^74^. Of note, 8 of the 12 taxa found to be significantly correlated with SE scores in patients, have been described to be part of the oral microbiome. While specific research investigating the oral microbiome with sleep behaviors is limited, some research has shown the translocation of oral microbes to the gut in people with colorectal cancer^75^. For example, specific oral bacteria, such as *Fusobacterium nucleatum (F. nucleatum*), *Parvimonas micra*, and *Peptostreptococcus stomatitis*, have been associated with CRC whereas the bacterium *F. nucleatum* has been shown to be associated with the development of tumor growth in CRC models^76–78^. Additionally, a review in 2019 discussed the link between various oral bacteria and their roles in different types of cancer including colorectal cancer^78^ and in 2020, a study investigated the origin of the bacterium *F. nucleatum* as originating in the oral microbiome and migrating to the gut microbiome of 7 people with CRC^79^. As the link between oral microbes and sleep behaviors has yet to be established, investigating the potential role of high sleep efficiency in lowering these oral bacteria and in CRC prognosis is warranted in future studies. While finding oral bacteria to be associated with sleep efficient in colorectal cancer patients is a somewhat unexpected finding, there has been research showing a link in people with colorectal cancer. This area could be beneficial for future research directions. This suggests the importance of exploring crosstalk between the oral and gut microbiomes, especially in patients with colorectal cancer, and could lead to new avenues for understanding how microbiome shifts might affect cancer outcomes, beyond the gut alone. Potentially, future research in this area could lead to new approaches for early detection or monitoring of colorectal cancer patients based on sleep patterns and microbiome composition.

Not only were oral microbiota correlated with SE scores among patients, but several were also found to be differentially abundant between dichotomized patients with low vs. high SE including: *Streptococcus mitis (S. mitis), Atopobium parvulum (A. parvulum), and Atopobium rimae (A. rimae). S. mitis*, a facultative anaerobe, is commonly found in the mouth and upper respiratory tract, where it plays a role in oral and gut interactions; imbalances with this bacterium may contribute to gastrointestinal issues^80^. While it aids in oral plaque formation, poor oral hygiene can lead to systemic inflammation, potentially impacting gut health through the gut-liver axis^80^. *A. parvulum*, an anaerobic bacterium in the gut, supports gut health by producing beneficial short-chain fatty acids like butyrate, which have anti-inflammatory effects and promote colonocyte energy^81^. This bacterium was found to be less abundant in our patients with higher SE. However, an overgrowth of *A. parvulum* or *A. rimae* (which while not as extensively documented as *A. parvulum*, is linked to similar gut dysbiosis and metabolic functions) could disrupt the gut microbiome, contributing to conditions like inflammatory bowel disease, though further research is needed to fully understand their roles in gastrointestinal diseases^82^. Overall, the findings in our study suggest that the oral microbiome plays a role in SE among CRC patients, potentially influencing not only oral and gut health but also broader aspects of systemic health. Given that the oral microbiota is intricately linked to both local (oral and gastrointestinal) and systemic inflammation, alterations in specific bacterial populations may serve as a pathway through which gut health impacts sleep patterns. The potential disruption of the gut microbiome by overgrowths of bacteria like *S. mitis*, *A. parvulum*, and *A. rimae* could contribute to dysbiosis, affecting immune responses, metabolism, and inflammatory processes that, in turn, might influence SE. However, while these associations provide valuable insights, the exact mechanisms linking oral microbiota to SE in CRC patients remain unclear. Further studies with larger sample sizes and more detailed analyses are essential to elucidate the specific microbial interactions and their clinical significance in sleep regulation, cancer progression, and overall health. Expanding research into this area could potentially lead to novel therapeutic approaches, where modulating the oral or gut microbiome may help improve both sleep quality and the general well-being of CRC patients.

Within this sample, a relatively low abundance of Bacteroidetes and a higher proportion of Actinobacteria compared to typical distributions reported in Western gut microbiome studies were observed. These differences might be partially explained by the demographic composition of the participant cohort, who self-identified as predominantly Hispanic (62.5%). Previous research has shown that gut microbial profiles vary significantly across racial and ethnic groups, with Hispanic individuals often exhibiting increased levels of Actinobacteria, particularly Bifidobacterium, and greater variability in Bacteroidetes abundance depending on dietary and lifestyle patterns^83,84^. Additionally, these findings could also be linked to the presence of colorectal cancer and any active cancer treatments that the patients were undergoing although no effect was found with the alpha diversity measures on treatment status. Though, cancer treatment may disrupt expected microbial signatures, particularly among the patients. These findings highlight the importance of considering race, ethnicity, and culturally specific dietary practices when interpreting gut microbiome composition, especially in clinical populations.

While the present study presents a novel exploration regarding the association of sleep with gut microbiome in patients with CRC and their sleep-partner caregivers, it is not without limitations. The current study used a cross-sectional analysis, which does not allow for causal interpretations. Longitudinal research would build on these findings and would allow for a broader understanding in investigating how gut health changes across cancer trajectories. Additionally, the relatively small sample size (n = 32; 16 dyads) limited our ability to statistically adjust for potential confounders in multivariable models. Although no significant differences were observed between patients and caregivers in demographic, dietary, or sleep indices, factors such as age, BMI, gender, and race/ethnicity are known to influence gut microbiome composition and may have contributed to the observed associations. Future studies with larger and more diverse samples will be needed to disentangle these effects and to control for key covariates. Other contributing environmental and lifestyle factors, such as stress and exercise levels, which were not examined in this study, should be considered in future work. Additionally, medication use and cancer treatment other than the chemotherapy type status of patients were not considered in the current analysis; previous research has shown interactions with pharmaceutical drugs with gut microbial features so this is an area that could potentially confound the findings in this work. Moreover, only three patients in our sample were undergoing active cancer treatment at the time of data collection, limiting our ability to draw meaningful conclusions about microbiome differences by treatment status. Thus, while no differences in alpha diversity were observed between treated and untreated patients, the small number of treated individuals prevents us from ruling out potential effects of cancer therapy on gut microbial composition. Future studies with larger cohorts and stratification by treatment status will be critical to better understand these relationships. Finally, the current participants did not have overly disrupted sleep, which limits the generalizability of findings. Furthermore, although self-reported sleep diaries are widely used to assess sleep patterns and efficiency, they have several limitations, including subjectivity, recall bias, and variability in participant compliance. Future research should explore the integration of self-reported data with objective measures, such as actigraphy, polysomnography, dim light melatonin onset (DLMO), and real-time ecological momentary assessment (EMA), to enhance the accuracy and reliability of sleep assessment.

Our findings revealed that although sleep behaviors were similar between the two groups, patients demonstrated differential associations between sleep efficiency and oral bacteria, with higher sleep efficiency associated with depletion of certain oral bacteria, a relationship not observed in caregivers. This finding suggests that the presence of CRC in patients may specifically modulate the relationship between sleep efficiency and oral bacteria. While caution is warranted due to the small sample size and cross-sectional design, this study provides valuable insights and may inform future research exploring the impact of sleep and other lifestyle behaviors on gut health in patient-caregiver dyads of colorectal cancer.

## Supplementary Information

Below is the link to the electronic supplementary material.

Supplementary Material 1

## Data Availability

FastQ microbiome sequence files are in the National Center for Biotechnology Information, Sequence Reads Archives data repository: Submission ID: SRP482831 and BioProject ID: PRJNA1063177 (https://www.ncbi.nlm.nih.gov/sra/?term=PRJNA1063177). All other data is available upon request, and we expect to make that data available for sharing with the scientific community once we have met our primary aims and outcomes. To protect subject privacy, access to the clinical data will be controlled by the Principal Investigator of the protocol (YK) and can be released upon request following the completion of a Data Sharing Agreement (DSA). The DSA will include instructions to: (1) use the data only for research purposes; (2) not identify any individual participant; (3) keep the data secured at all times; and (4) destroy or return the data after analyses have been completed.

## Abbreviations

CRC: Colorectal cancer
SE: Sleep efficiency
WASO: Wake after sleep onset
SOL: Sleep onset latency
LFC: Log fold change
CLR: Centralized log ratio
