# Supplementary material for: Associations between sleep and the gut microbiome in adults with colorectal cancer and their caregivers
Source: Sci Rep. 2025 Sep 22;15:32624. doi: 10.1038/s41598-025-18402-2 (PMC12454654; doi:10.1038/s41598-025-18402-2)
Supplement: Supplementary file 1 — Supplementary Material 1 [file 41598_2025_18402_MOESM1_ESM.docx]

**Associations Between Sleep and the Gut Microbiome in Adults with Colorectal Cancer and Their Caregivers**

Jennifer J. Barb^1^, Lena J. Lee^1*^, Ayaan Ahmed^1^, Elisa H. Son^1^, Shubhi Nanda^1^, Li Yang^1^, Yuguang Ban^2^, Amanda Ting^2^, Thomas C. Tsai^2^, Youngmee Kim^2^

**Supplemental Material**

**Supplemental Table 1: Dietary intake indices between patients and caregivers
Supplemental Table 2: Alpha Diversity Measures by Treatment Status in patients only
Supplemental Table 3: Alpha Diversity Measures by Cancer Stage in Patients only
Supplemental Table 4: Alpha Diversity Measures by Diet Quality and Fiber Intake in patients and caregivers
Supplemental Table 5: Differentially abundant gut microbial taxa between patients and caregivers
Supplemental Table 6: Differentially abundant microbial genomic potential (KEGG) pathways between patients and caregivers
Supplemental Table 7:** **Microbial genomic function potential significant correlations with sleep measures within patients and caregivers
Supplemental Table 8: Differential alpha diversity measures within patients and caregivers with high and low sleep efficiency
Supplemental Table 9: Differential taxa in caregivers with high and low sleep efficiency
Supplemental Figure 1A: Relative abundances of phyla across patients and caregivers
Supplemental Figure 1B: Relative abundances of species across patients and caregivers
Supplemental Figure 2: KEGG pathway genomic functional potential differences between patients and caregivers
Supplemental Figure 3: Taxa within caregivers that were significantly different between high and low sleep efficiency
Supplemental Figure 4: Bivariate plots showing significant taxa correlated with sleep efficiency values in patients**

**Supplemental Tables:**

**Supplemental Table 1: Dietary intake indices between patients and caregivers**

| **Dietary Intake** | | | | | |
| --- | --- | --- | --- | --- | --- |
| **Dietary Measures** | **All N=33** | **Patients**  **N = 17** | **Caregivers**  **N = 16** | ***Statistic*** | ***P*-value**^a,b^ |
|  | Mean (SD) | | |  |  |
| HEI-2020 | 56.96 (13.21) | 58.76 (14.98) | 55.05 (11.20) | S = 13.00 | .528 |
| Total Energy (kcal) | 1818.71 (796.85) | 1872.91 (884.26) | 1761.11 (716.72) | S = 1.00 | .979 |
| Total Energy (excluding alcohol) (kcal) | 1771.05 (776.98) | 1834.84 (863.60) | 1703.28 (694.87) | S = 1.00 | .860 |
| % kcal from Carbohydrates | 47% (9) | 48% (9) | 45% (10) | S = 22.00 | .274 |
| % kcal from Fat | 33% (6) | 32% (6) | 34% (6) | S = -18.00 | .375 |
| % kcal from Protein | 18% (6) | 19% (7) | 18% (5) | S = -2.00 | .934 |
| % kcal from Alcohol | 2% (5) | 2% (5) | 3% (5) | S = -1.50 | .844 |
| Total Alcohol (g) | 6.81(17.80) | 5.44 (19.90) | 8.26 (15.78) | S = -1.50 | .844 |
| Total Carbohydrate (g) | 206.78 (94.89) | 219.98 (108.23) | 192.75 (79.41) | S = 13.00 | .528 |
| Total Fiber (g) | 16.66 (10.20) | 18.61 (11.78) | 14.59 (8.07) | S = 33.00 | .093 |
| Total Protein (g) | 81.78 (35.26) | 83.97 (36.23) | 79.46 (35.22) | S=5.00 | .829 |
| Total Fat (g) | 68.53 (35.11) | 68.78 (38.23) | 68.27 (32.72) | S= -8.00 | .705 |

Note. ^a^Wilcoxon-Signed Rank test; ^b^one patient sample not included in testing procedure because dietary intake of paired caregiver is missing.

**Supplemental Table 2: Alpha Diversity Measures by Treatment Status in patients only**

| **Active Cancer Treatment Status for Patients** | | | |
| --- | --- | --- | --- |
| **Alpha Diversity Measures** | **No**  **N = 16*** | **Yes**  **N = 3** | ***P*-value^a^** |
| Mean (SD), Range | | | |
| Shannon | 2.79 (.46), 1.92 – 3.26 | 2.89 (.42), 2.62 – 3.38 | .696 |
| Inverse Simpson | 9.62 (3.81), 4.15 – 15.24 | 11.36 (5.32), 7.85 – 17.48 | .654 |
| Chao1 | 154.46 (18.59), 119.50 – 178.50 | 158.17 (30.35), 126.50 – 187.00 | .823 |

Note. ^a^Mann-Whitney U Test; ^*^unknown whether one patient on cancer treatment

**Supplemental Table 3: Alpha Diversity Measures by Cancer Stage in Patients only**

| **Cancer Stage** | | | | | |
| --- | --- | --- | --- | --- | --- |
| **Alpha diversity Measures** | **I**  **N = 6** | **II**  **N=3** | **III**  **N = 9** | **IV**  **N = 2** | ***P*-value^a^** |
| Mean (SD), range | | | | | |
| Shannon | 2.96 (.36), 2.30 – 3.26 | 3.15 (.08), 3.06 – 3.23 | 2.60 (.51),  1.92 – 3.38 | 2.74 (.07),  2.69 – 2.79 | .210 |
| Inverse Simpson | 10.93 (3.51), 5.57 – 15.24 | 12.31 (1.63), 10.79 – 14.04 | 8.47 (4.62), 4.15 – 17.48 | 9.38 (.91),  8.74 – 10.02 | .328 |
| Chao1 | 158.33 (20.28), 119.50 – 174.50 | 168.00 (9.84), 159.00 – 178.50 | 150.83 (21.08), 126.50 – 187.00 | 146.75 (20.15), 132.50 – 161.00 | .436 |

Note^. a^Kruskal-Wallis Test

**Supplemental Table 4: Assessing Alpha Diversity Measures by Diet Quality and Fiber Intake**

| **Patient or Caregiver** | **Diet Variable** | **Alpha Diversity Variable** | **Spearman ρ** | **P-value^a^** |
| --- | --- | --- | --- | --- |
| CG | HEI-2020 | Shannon | 0.118 | 0.519 |
| CG | HEI-2020 | Chao1 | -0.020 | 0.909 |
| CG | HEI-2020 | Inverse Simpson | 0.138 | 0.451 |
| PT | HEI-2020 | Shannon | 0.464 | 0.006* |
| PT | HEI-2020 | Chao1 | 0.148 | 0.403 |
| PT | HEI-2020 | Inverse Simpson | 0.405 | 0.018* |
| CG | Fiber.day.g | Chao1 | -0.047 | 0.800 |
| CG | Fiber.day | Chao1 | 0.005 | 0.978 |
| CG | Fiber.day | Inverse Simpson | -0.039 | 0.831 |
| PT | Fiber.day.g | Chao1 | 0.081 | 0.659 |
| PT | Fiber.day | Chao1 | -0.22 | 0.220 |
| PT | Fiber.day | Inverse Simpson | -0.006 | 0.975 |

Note. ^a^Spearman Correlation Test; *=p<.05

**Supplemental Table 5: Differentially abundant gut microbial taxa between patients and caregivers**

| **Species** | **Log Fold Change (LFC) of PT-CG** | **Patients**  **CLR**  **N=20** | **Caregivers CLR**  **N=20** | **Statistic** | **P-value^a^** | **FDR** |
| --- | --- | --- | --- | --- | --- | --- |
| *Coprococcus catus* | -4.87 | -1.88 (4.80) | 2.99 (2.87) | S= 189.00 | <.001 | 6% |
| *Gemmiger formicilis* | -3.85 | -1.04 (4.05) | 2.81 (2.72) | S=194.00 | <.001 | 4% |
| *Lachnospira pectinoschiza* | -3.58 | -3.57 (4.49) | .01 (3.95) | S= 170.00 | .014 | 16% |
| *Bacteroides thetaiotaomicron* | -3.08 | -1.51 (4.43) | 1.57 (1.69) | S=171.00 | .012 | 16% |
| *Blautia obeum* | -1.73 | 3.16 (2.56) | 4.88 (1.61) | S=175.00 | <.001 | 13% |
| *Faecalibacterium prausnitzii* | -1.66 | 4.62 (2.54) | 6.28 (1.21) | S=171.00 | .012 | 16% |
| *Actinomyces naeslundii* | 2.60 | .03 (2.57) | -2.57 (2.99) | S=28.00 | <.001 | 9% |
| *Bifidobacterium bifidum* | 2.79 | 1.87 (3.77) | -.92 (4.18) | S=35.00 | <.001 | 13% |
| *Turicibacter sanguinis* | 3.11 | -.68 (3.79) | -3.79 (3.68) | S=40.00 | .014 | 16% |
| *Blautia sp CAG 257* | 3.17 | 2.49 (4.09) | -0.68 (3.67) | S=32.00 | <.001 | 13% |
| *Pediococcus acidilactici* | 3.52 | -.97 (5.13) | -4.48 (3.45) | S=43.00 | .019 | 20.6% |
| *Clostridium clostridioforme* | 3.74 | -.47 (5.25) | -4.20 (4.29) | S=27.00 | <.001 | 9% |
| *Blautia coccoides* | 5.22 | 1.19 (4.89) | -4.02 (4.95) | S=33.00 | <.001 | 13% |

Note. ^a^Wilcoxon signed rank test; Abbreviations: CLR= Central Log Ratio, FDR= False Discovery Rate, PT=patient, CG=caregiver

**Supplemental Table 6: Differentially abundant microbial genomic potential (KEGG) pathways between patients and caregivers**

| **KEGG Pathway** | **Log Fold Change of PT-CG** | **Patients Log_10_ N=20** | **Caregivers Log_10_ N=20** | **Statistic** | **P-Value^a^** | **FDR** |
| --- | --- | --- | --- | --- | --- | --- |
| PWY-6737: starch degradation V | -.11 | 3.88 | 3.99 | S= 313.00 | .009 | 69% |
| TRNA-CHARGING-PWY: tRNA charging | -.09 | 3.77 | 3.86 | S=317.00 | .012 | 69% |
| NONMEVIPP-PWY: methylerythritol phosphate pathway I | -.08 | 3.79 | 3.86 | S= 321.00 | .017 | 69% |
| PWY-5464: superpathway of cytosolic glycolysis | .34 | 2.21 | 1.87 | S=465.00 | .018 | 69% |
| PWY0-1319: CDP-diacylglycerol biosynthesis II | -.05 | 3.79 | 3.85 | S= 328.00 | .028 | 69% |
| PWY-5667: CDP-diacylglycerol biosynthesis I | -.05 | 3.79 | 3.85 | S= 328.00 | .028 | 69% |
| PWY-5941: glycogen degradation II | -.48 | 2.38 | 2.86 | S= 329.00 | .029 | 69% |
| SALVADEHYPOX-PWY: adenosine nucleotides degradation II | -.13 | 3.21 | 3.35 | S= 330.00 | .032 | 69% |
| PWY-5367: petroselinate biosynthesis | -.55 | 2.49 | 3.04 | S= 331.00 | .034 | 69% |
| P124-PWY: Bifidobacterium shunt | -.37 | 2.46 | 2.83 | S= 142.00 | .034 | 69% |
| PWY-6163: chorismate biosynthesis from 3-dehydroquinate | -.06 | 3.89 | 3.95 | S= 332.00 | .036 | 69% |
| PWY-5101: L-isoleucine biosynthesis II | -.44 | 2.44 | 2.88 | S= 333.00 | .039 | 69% |
| PWY-5177: glutaryl-CoA degradation | -.43 | 2.66 | 3.09 | S= 335.00 | .044 | 69% |

Note. ^a^Wilcoxon Signed Rank Test; Abbreviations: FDR= False Discovery Rate. KEGG pathways are derived from the KEGG database (43).

**Supplemental Table 7:** **Microbial genomic function potential significant correlations with sleep measures within patients and caregivers**

| **Significant Correlations between Sleep Measures** | | | | |
| --- | --- | --- | --- | --- |
| **Sleep measure** | **KEGG Pathway** | **ρ** | **P-Value^a^** | **FDR** |
| **Caregiver** | | | | |
| **Sleep Onset Latency** | PWY-5845: superpathway of menaquinol-9 biosynthesis | -.807 | <.001 | **4%** |
|  | PWY-5896: superpathway of menaquinol-10 biosynthesis | -.807 | <.001 | **4%** |
| **Time in Bed** | PWY-6590: superpathway of Clostridium acetobutylicum acidogenic fermentation | -.734 | <.001 | **5%** |
| **Patient** | | | | |
| **Time In Bed** | PWY-6588: pyruvate fermentation to acetone | -.167 | <.001 | **5%** |
|  | CENTFERM-PWY: pyruvate fermentation to butanoate | -.734 | <.001 | **5%** |

Note. ^a^Spearman Correlation Test; KEGG pathways are derived from the KEGG database (43).

**Supplemental Table 8: Differential alpha diversity measures within patients and caregivers with high and low sleep efficiency**

| **High/ Low Sleep Efficiency** | | | | |
| --- | --- | --- | --- | --- |
|  | **Patient** | | **Caregiver** | |
| **Alpha Diversity Measure** | **Z statistic** | **P-value** | **Z statistic** | **P-value** |
| Inverse Simpson | -2.35 | **.019** | .000 | 1.000 |
| Chao1 | -.578 | .563 | -1.048 | .295 |
| Shannon | -2.103 | **.035** | -0.436 | .663 |

*Mann-Whitney U test. Within patient and caregiver alpha diversity comparisons between high/low sleep efficiency groups for patients and caregivers

**Supplemental Table 9: Differential taxa in caregivers with high and low sleep efficiency**

| **Caregiver High/Low Sleep Efficiency** | | | |
| --- | --- | --- | --- |
| **Species** | **Z statistic** | **P-Value^a^** | **FDR** |
| *Alistipes shahii* | 2.18 | .029 | 81% |
| *Bacteroides ovatus* | 2.53 | .011 | 81% |
| *Clostridium clostridioforme* | 2.01 | .045 | 81% |
| *Mogibacterium diversum* | -2.18 | .029* | 81% |

^a^Mann-Whitney U test. Within caregiver taxa abundance comparisons between high/low sleep efficiency groups. Unadjusted differential taxa (p<.05 and >20%FDR).

**Supplemental Figures:**

**Supplemental Figure 1: Relative abundances of phyla and species across patients and caregivers**


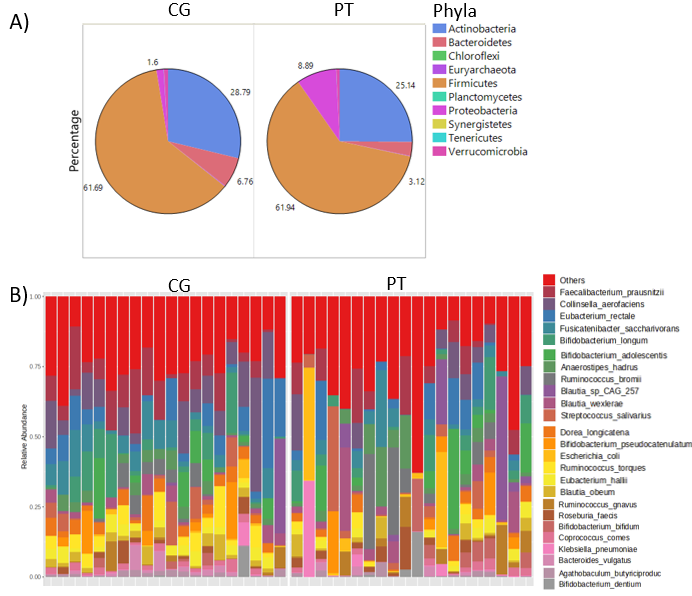


**Legend**: Average gut microbiome features of adults with CRC and caregivers. A) Proportions of bacterial phyla making up the gut microbiomes of caregivers and patients B) Relative abundances of bacterial species distributed across the gut microbiomes of caregivers and patients.

**Supplemental Figure 2: KEGG pathway genomic functional potential differences between patients and caregivers**


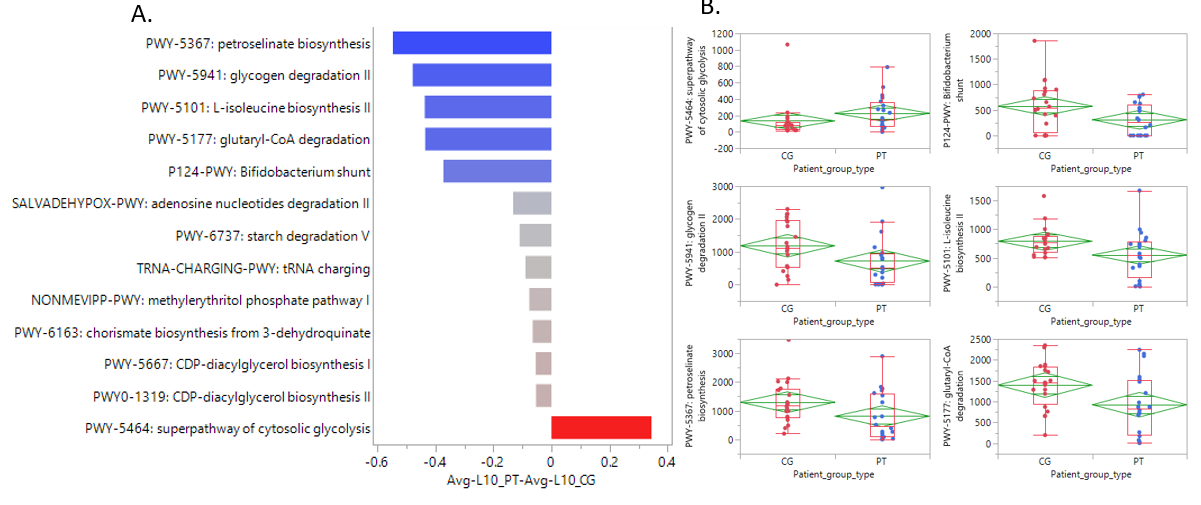


**Legend**: A) Thirteen KEGG pathways were found to be differentially abundant (p<.05 uncorrected) (Wilcoxon rank sum test). Comparison between CG and PT three pathways that were more abundant in CG ( starch degradation V (*p*=.009), tRNA charging (*p*=.012), methylerythritol phosphate pathway I (*p*=.017), superpathway of cytolosic glycolysis (*p*=.018), CDP-diacylglycerol biosynthesis II (*p*=.028), CDP-diacylglycerol biosynthesis I (*p*=.028), glycogen degradation II (*p*=.029), adenosine nucleotides degradation II (*p*=.032), petroselinate biosynthesis (*p*=.034), Bifidobacterium shunt (*p*=.034), chorismite biosynthesis from 3-dehydryoquinate (*p*=.036), L-isoleucine biosynthesis II (*p*=.039), glutaryl-CoA degradation (*p*=.044) were all found to be differentially abundant between patients and caregivers. B) Box plot of 6 differential genomic potential KEGG pathways. Comparison between CG and PT one pathway that was more abundant in PT, superpathway of cytolosic glycolysis (*p*=.018), and five that were more abundant in CG, glycogen degradation II (*p*=.029), petroselinate biosynthesis (*p*=.034), Bifidobacterium shunt (*p*=.034), L-isoleucine biosynthesis II (*p*=.039), and glutaryl-CoA degradation (*p*=.044).

**Supplemental Figure 3: Bivariate plots showing significant taxa correlated with sleep efficiency values in patients**

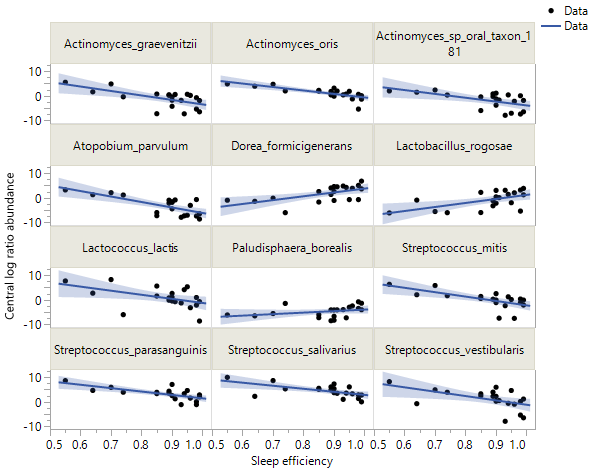


**Legend**: Bivariate linear regression plot showing gut microbial species within patients that significantly correlated with patient sleep efficiency scores at *p*<.05 (20%FDR). Comparison between SE scores in patients showing species *Actinomyces graevenitzii* (*p*<.001), *Actinomyces oris* (*p*<.001), *Actinomyces sp oral taxon 181* (*p*<.001), *Atopobium parvulum* (*p*<.001), *Dorea formicigenerans* (*p*<.001), *Lactobacillus rogosae* (*p*<.001), *Lactococcus lactis* (*p*=.016), and *Paludisphaera borealis* (*p*=.016).*Streptococcus mitis* (*p*<.001), *Streptococcus parasanguinis* (*p*<.001), *Streptococcus salivarius* (*p*<.001), *Streptococcus vestibularis* (*p*<.001).

**Supplemental Figure 4: Taxa within caregivers that were significantly different between high and low sleep efficiency**


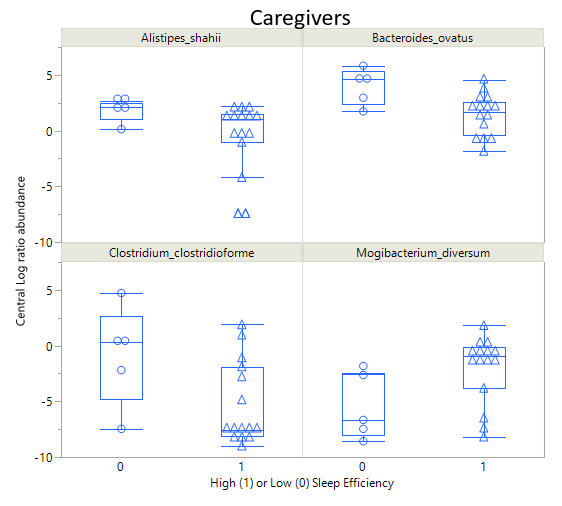


**Legend**: Box plot showing the four taxa with significantly different abundances between caregivers with high and low sleep efficiency score (FDR>20%). Significance p<.05 unadjusted. Comparison between high and low SE in caregivers showing species *Alistipes shahii (p=*0.029), *Bacteroides ovatus* (*p*=.011), *Clostridium clostridioforme* (*p*=.045), Mogibacterium *diversum (p*=.029).
